# Left ventricular electromechanical dyssynchrony and mortality in cardiothoracic intensive care

**DOI:** 10.1186/cc12110

**Published:** 2013-03-19

**Authors:** G Tavazzi, M Bojan, A Duncan, A Vazir, S Price

**Affiliations:** 1 University of Pavia Foundation Policlinico San Matteo IRCCS, Pavia, Italy; 2 Necker-Enfants Malades University Hospital, Paris, France; 3 Royal Brompton Hospital, London, UK

## Introduction

Global left ventricular electromechanical dyssynchrony (GLVD) is uncoordinated LV contraction that reduces the extent of intrinsic energy transfer from the myocardium to the circulation leading to a reduction in peak LV pressure rise, prolonged total isovolumic time (t-IVT) and fall in stroke volume [1]. This potentially important parameter is not routinely assessed in critically ill cardiothoracic patients.

## Methods

A prospective analysis of retrospectively collected data in cardiothoracic ICU patients who underwent echocardiography was performed. In addition to epidemiological factors, echo data included comprehensive assessment of LV/RV systolic and diastolic function including Doppler analysis of isovolumic contraction/ relaxation, ejection time (ET) and filling time (FT). t-IVT was calculated as (60 - (total ET + total FT)) and the Tei Index as (ICT + IRT) / ET. t-IVT >14 second/minute and Tei index >0.48 were used to define GLVD [2]. Data are shown as mean ± SD/median (interquartile range).

## Results

A total of 103 patients (63.5 ± 18.4 years), 65 male (63%), APACHE II score (14.6 ± 7.4) were included. The prevalence of GLVD was high (24/103, 22%) and associated with significantly increased mortality, 7.5% vs. 25% (*P *= 0.02). There was no difference in requirement for cardiorespiratory support between the two populations, but there were significant differences (no GLVD vs. GLVD) in requirement for pacing (35% vs. 62%, *P *= 0.02), atrial fibrillation (20% vs. 41%, *P *= 0.03), QRS duration (92.0 (80.0 to 120.0) vs. 116.5 (95.0 to 154.0), *P *= 0.01) and QTc (460.0 (416.0 to 498.5) vs. 477.5 (451.2 to 541.0), *P *= 0.02). There was no significant difference in ejection fraction (no GLVD 43.0 (35.0 to 49.5) vs. GLVD 39.6 (29.5 to 49.7), *P *= 0.43), mitral regurgitation (40.5% vs. 62.5%, *P *= 0.06), or any other measures of LV systolic or diastolic function between the two groups. There was good correlation between the two methods used to assess dyssynchrony (LV t-IVT:LV Tei index correlation coefficient = 0.80, *P <*0.001).

## Conclusion

GLVD that limits cardiac output is common in the cardiothoracic ICU, and significantly related to mortality. When diagnosed, the underlying cause should be sought and treatment instigated to minimize the t-iVT (pacing optimization/revascularization/ inotrope titration/volaemia optimization).
